# H_2_O_2_/pH Dual-Responsive Biomimetic Nanoenzyme Drugs Delivery System for Enhanced Tumor Photodynamic Therapy

**DOI:** 10.1186/s11671-022-03738-9

**Published:** 2022-10-29

**Authors:** Xinyuan Li, Qing Ji, Chao Yan, Ziyu Zhu, Zhihui Yan, Ping Chen, Yisen Wang, Li Song

**Affiliations:** 1 The Affiliated Huai’an Hospital of Xuzhou Medical University and The Second People’s Hospital of Huai’an, No.62, Huaihai Road (S.), Huai’an, 223002 China; 2 YanCheng NO.1 People’s Hospital, Yancheng, 224001 China; 3 School of Medicine, Jiangsu University, Zhenjiang, 212013 China; 4 Institute of Translational Medicine, Medical College, Yangzhou University, Yangzhou, 225009 China

**Keywords:** Phototherapy, Catalase-like activity, Tumor microenvironment, Nano-platform

## Abstract

Phototherapy has been recognized as a photochemical process to treat tumor via induce cancer cells necrosis and death, with minimal invasiveness, higher selectivity, and few side effects. However, the therapy effects of phototherapy are often compromised by the hypoxia, high levels of hydrogen peroxide, and glutathione of tumor microenvironment (TME). Therefore, we constructed a catalase-like activity bionic metal–organic framework drugs delivery system (FA-EM@MnO_2_/ZIF-8/ICG) with tumor microenvironment controllable releasing. In this system, photosensitizer indocyanine green (ICG) was introduced into zeolite imidazole salt skeleton 8 (ZIF-8) by one-step methods, forming ZIF-8/ICG nano-platform, which can effectively avoid ICG-induced phototoxicity and aggregation-induced quenching during transport. MnO_2_ with catalase-like activity was coated on the surface of ZIF-8/ICG nano-platform, which made it have the ability of self-supplying O_2_ under the condition of H_2_O_2_ in TME. Exposure under near-infrared light can alleviate the anoxic TME, thus improving the phototherapy efficiency. In addition, folate-functionalized erythrocyte membrane is coated on the surface of MnO_2_/ZIF-8/ICG, which can endow FA-EM@MnO_2_/ZIF-8/ICG with the ability of targeted drug administration and immune elimination avoidance. Therefore, FA-EM@MnO_2_/ZIF-8/ICG nano-platform has the catalase-like activity, which can alleviate the oxidative stress state of TME and provide a beneficial environment for photodynamic therapy of tumor.

**Supplementary Information:**

The online version contains supplementary material available at 10.1186/s11671-022-03738-9.

## Introduction

Tumor is one of the major diseases that threatens human health and causes millions of deaths every year [1]. Chemotherapy and surgical resection are still the major intervention approach in clinical for tumor therapy nowadays [2, 3]. However, surgical resection and chemotherapy suffered from the high risk of tumor recurrence and caused serious damage to normal organs and tissues. In order to enhance tumor therapy effects and avoid unnecessary side effects to normal tissues, various novel tumor therapy approaches have been developed, such as starvation therapy (ST), photothermal therapy (PTT), photodynamic therapy (PDT), and sonodynamic therapy (SNT) [4–6].

Due to tumor tissues lacking autonomous regulatory ability leading tumor tissues to be a relative heat reservoir, thus PTT and PDT have the inherent advantages in the field of tumor therapy [7, 8]. However, cancer cells grown fast as well as distorted blood vessels would lead tumor tissues lack adequate oxygen supply, in the state of oxidative stress as well as acidification condition leading tumor therapy effects are compromised [9]. In addition, using PTT alone may cause serious damage to surround tissues and organs, because cancer cells need high temperature to kill [10].

With the development of biomedical, various kinds of photothermal material and photosensitizers (PSs) have been widely applied in the tumor therapy. Among them, indocyanine green (ICG), as the ideal medical diagnostic agent, is approved by Food and Drug Administration (FDA) due to its low toxicity [11]. In addition, ICG has strong fluorescence characteristics and near-infrared absorption ability, which can penetrate into deep tissues, so that ICG can be applied to photothermal therapy triggered by near-infrared light (NIR) guided by fluorescence imaging [12, 13]. Nevertheless, ICG is inevitable distributed around the entire body, especially in skin, and also causes phototoxicity to them after injection [2]. What’s more, ICG still has aggregation-induced quenching phenomenon in the process of blood circulation, which will lead to low photothermal conversion efficiency, thus reducing the therapeutic efficiency [14]. Various strategies have been developed to overcome these drawbacks associated with ICG, but the result is disappointing.

According to literature reports, metal–organic frameworks (MOFs) have a large surface area, finely tunable pore shape and size, excellent biodegradability, and the surface is easily modified, which has been widely applied in the field of biomedical, especially as drugs delivery system for disease therapy [15, 16]. As drug delivery system, MOFs have many incomparable advantages, but their application is widely restricted by some prominent problems, such as difficulty to achieve on-demand release, easy to be cleared by body’s immune system, and unsatisfactory targeted delivery capability [17–19]. Zeolite Imidazole Skeleton 8 (ZIF-8), as the typical MOFs, is easily decomposed in acid tumor microenvironment (TME), because their metal–ligand interaction is very unstable [20]. Especially, the synthesis progress of ZIF-8 is very mild, which can effectively maintain the biological activity of drugs, the surface of ZIF-8 is easy to be modified to improve its biocompatibility and tumor treatment effects at the same time [21].

**Scheme 1:**
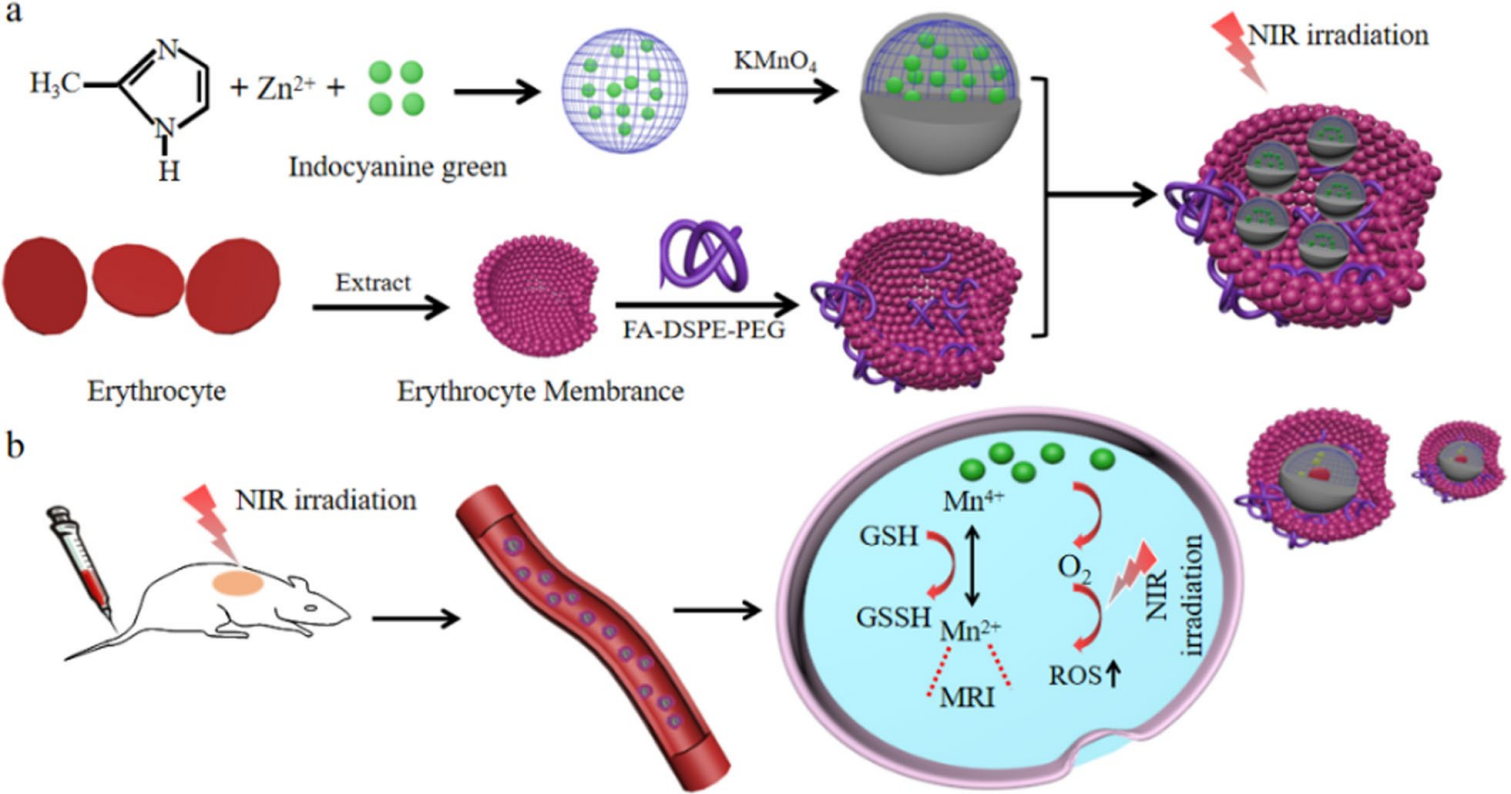
Illustration for the preparation of a FA-EM@MnO_2_/ZIF-8/ICG nano-platform for MRI imaging-guided cancer therapy. (**a**) The construction of FA-EM@MnO_2_/ZIF-8/ICG nano-platform through drug loading, MnO_2_ decoration and erythrocyte membrane coating. (**b**) Proposed action mechanism of FA-EM@MnO_2_/ZIF-8/ICG nano-platform in a mouse tumor model

In summary, we constructed environment-responsive drugs nano-platform based on ZIF-8 (FA-EM@MnO_2_/ZIF-8/ICG) with catalase-like activity for photothermal therapy of tumors. As shown in Scheme 1, FA-EM@MnO_2_/ZIF-8/ICG was prepared by biomimetic mineralization and coprecipitation reaction between Zn^2+^ ion and 2-methylimidazole to load ICG (ZIF-8/ICG), whose surface was coated with a layer of MnO_2_ (MnO_2_/ZIF-8/ICG), and then MnO_2_/ZIF-8/ICG exterior surface was further cloaked with folic acid modified erythrocyte membrane (FA-EM) (Scheme 1a). Thanks to the fact that manganese dioxide has nano-enzyme-like activity, the obtained FA-EM@MnO_2_/ZIF-8/ICG can alleviate the oxidative stress of tumor tissues by catalyzing and decomposing endogenous H_2_O_2_ into O_2_, and correspondingly reduce the hypoxia condition of tumor to improve the results of photodynamic therapy (Scheme 1b) [22]. Meanwhile, folic acid-functionalized erythrocyte membrane was further coated into the surface of MnO_2_/ZIF-8/ICG, which can endow FA-EM@MnO_2_/ZIF-8/ICG targeted delivery to the tumor tissues via folic acid receptors, and avoid eliminated by body immune system to prolong their blood circulation [23]. Therefore, our designed FA-EM@MnO_2_/ZIF-8/ICG nano-platform can realize the controlled release of ICG in time and space. By simultaneously targeting delivery, avoiding elimination by body immune system, continuously supplying O_2_, ameliorating oxidative stress, and overcoming hypoxia TME. Thus, our FA-EM@MnO_2_/ZIF-8/ICG nano-platform provided an instructive TME for improving tumor PDT.

## Experimental Section

### Synthesis of FA-EM@MnO_2_/ZIF-8/ICG

Erythrocyte membrane (EM) vesicles were obtained according to the previous methods with some modification [24]. Firstly, erythrocytes were collected from BALB/c mice fresh blood and washed with PBS for several times to remove plasma and unwanted cells until the supernatant became colorless. Secondly, the obtained erythrocytes were immersed into 5 mL ultrapure water at 4 °C for 2 h, during that the intracellular components inside of the erythrocytes were released. Thirdly, the products were washed with PBS for three times and followed by extrusion through polycarbonate membranes. Lastly, EM was further dispersed in PBS solution and stored at – 80 °C before used.

To obtain the final products FA-EM@MnO_2_/ZIF-8/ICG. DSPE-PEG-FA (10 mg) was added into the EM solution and continuous stirred at 4 °C for 12 h [25]. Then, the products FA modified EM (FA-EM) was collected by centrifuging (15,000 rpm, 5 min) and washed with PBS for three times. Finally, the obtained FA-EM was mixed with MnO_2_/ZIF-8/ICG followed by stirring at 1000 rpm for 24 h; and then the products were washed with PBS for three times and centrifuged at 4000 rpm to obtain FA-EM@MnO_2_/ZIF-8/ICG nano-platform [26].

### Evaluation of O_2_ Generation

The concentration of extracellular oxygen, which was generated in the FA-EM@MnO_2_/ZIF-8/ICG was quantified by a dissolved oxygen meter [27]. Briefly, FA-EM@MnO_2_/ZIF-8/ICG dispersion was put into 50-mL beaker, and H_2_O_2_ (100 µL, 30 mM) was added. Then, the dissolved oxygen meter probe was inserted into the mixing solution to detect oxygen concentration with continued stirring. Then, PBS, H_2_O_2_ solution, ZIF-8/ICG + H_2_O_2_, and MnO_2_/ZIF-8/ICG + H_2_O_2_ were applied as control. Additionally, O_2_ generation within the cells was monitored through RDPP, whose red fluorescence signal can be quenched by O_2_. Briefly, 4T1 cells were incubated with 10 µM RDPP for 4 h at 37 °C. Then, 4T1 cells were treated with PBS, H_2_O_2_, FA-EM@MnO_2_/ZIF-8/ICG, and FA-EM@MnO_2_/ZIF-8/ICG + H_2_O_2_, and incubated for another 4 h. Finally, 4T1 cells were washed with PBS for three times and observed through fluorescence microscopy [28].

### Releasing Behavior of ICG

To investigate the ICG releasing behavior from FA-EM@MnO_2_/ZIF-8/ICG nano-platform, 10 mg FA-EM@MnO_2_/ZIF-8/ICG nano-platform was immersed into normal physiological environment (pH = 7.4), pH = 5.5, 10 mM GSH, 30 μM H_2_O_2_ and simulate tumor microenvironment (TEM, pH = 5.5, 10 mM GSH, 30 μM H_2_O_2_), respectively. The different FA-EM@MnO_2_/ZIF-8/ICG dispersion solution were incubated at 37 °C on a horizontal shaker with 300 rpm. At designed points, 3 mL supernatant solution was withdrawn and the ICG released content was determined by fluorescence spectroscopic instrument [29, 30].

### Photothermal Performance Measurements

The FA-EM@MnO_2_/ZIF-8/ICG dispersion solution (ICG concentration: 150 and 200 μg/mL), and MnO_2_/ZIF-8/ICG (ICG concentration: 200 μg/mL) were exposed under 808 nm NIR irradiation with 1.5 W/cm^2^ or 2.0 W/cm^2^ for 600 s and recorded by infrared thermal image, respectively. PBS solution was set as control group [5, 31].

### Detection of Reactive Oxygen Species

DPBF as the chemical probe was utilized to detect the reactive oxygen species (ROS) generation of FA-EM@MnO_2_/ZIF-8/ICG nano-platform with different treatment in extracellular, which could react with DPBF to lead an irreversible reduction in the DPBF absorbance [32, 33]. Typically, FA-EM@MnO_2_/ZIF-8/ICG and DPBF were, respectively, added into 2 mL DPBF/DMSO (1:1) and exposed under 808 nm NIR irradiation (2.0 W/cm^2^). At the designed time point, the absorbance of DPBF near 417 nm was recorded by UV–visible spectrophotometer. The absorbance value of DPBF in H_2_O_2_ solution, FA-EM@MnO_2_/ZIF-8/ICG + NIR, and FA-EM@MnO_2_/ZIF-8/ICG + H_2_O_2_ were also recorded as the control.

Besides, ROS generation within the cells was monitored through a cell-permeable dye-DCFH-DA. As well all know, DCFH-DA was nonfluorescent signal at normal condition, which can be oxidized into stronger green fluorescent 2,7-dichlorofluorescin (DCF) by ROS [34]. First, 4T1 cells were seeded into 12-well plate and treated with different approaches (blank, NIR irradiation, H_2_O_2_, FA-EM@MnO_2_/ZIF-8/ICG + NIR, FA-EM@MnO_2_/ZIF-8/ICG + H_2_O_2_, and FA-EM@MnO_2_/ZIF-8/ICG + H_2_O_2_ + NIR. After 6 h incubation, the old medium was discarded and supplanted with fresh medium and exposed under 808 nm NIR irradiation with the power of 2.0 W/cm^2^ for 10 min. Then, the chemical probe of DCFH-DA was added and incubated for 30 min. Lastly, 4T1 cells were washed with PBS for three times and detected by fluorescence microscopy.

### IN VIVO Therapeutic Effects

5 × 10^6^ 4T1 cells were injected into the right back of BALB/c female mice to establish the subcutaneous 4T1 tumor model [35]. All animal experiments were approved according to the Institutional Animal Care and Use Committee of Yangzhou University. When the tumor volume reached for about 50 mm^3^, the mice were injected intravenously (i.v.) with PBS or different kinds nano-platform (15 mg nano-platform/kg that for about equal the 10 mg ICG/kg) once every 3d for a total of five times accompanied with NIR irradiation [29]. The mice were randomly divided into five groups (*n* = 6) as the following methods: (1) PBS, (2) PBS + NIR, (3) ZIF-8/ICG + NIR, (4) MnO_2_/ZIF-8/ICG + NIR, (5) FA-EM@MnO_2_/ZIF-8/ICG + NIR. For mice that received photothermal therapy, at 24 h after each injection the mice were exposed under 808 nm NIR irradiation at 2.0 W/cm^2^ for 10 min. Tumor volume and body weight of each mouse were monitored every 3 days during the whole assays. After 15 d treatment, the mice were euthanized for further investigation. The tumor volume was evaluated as following formula: Tumor volume (mm^3^) = 1/2 × width^2^ × length [36].

### Statistical Analysis

All the experiment data were analyzed by OriginPro and SPSS.17.0. All the error bars indicated mean ± standard deviation (mean ± SD). The Student’s *t* test was applied for statistical analysis. The *p* value of < 0.05 was considered statistically significant. *p < 0.05, **p < 0.01, ***p < 0.001.

## Results and Discussion

**Fig. 1:**
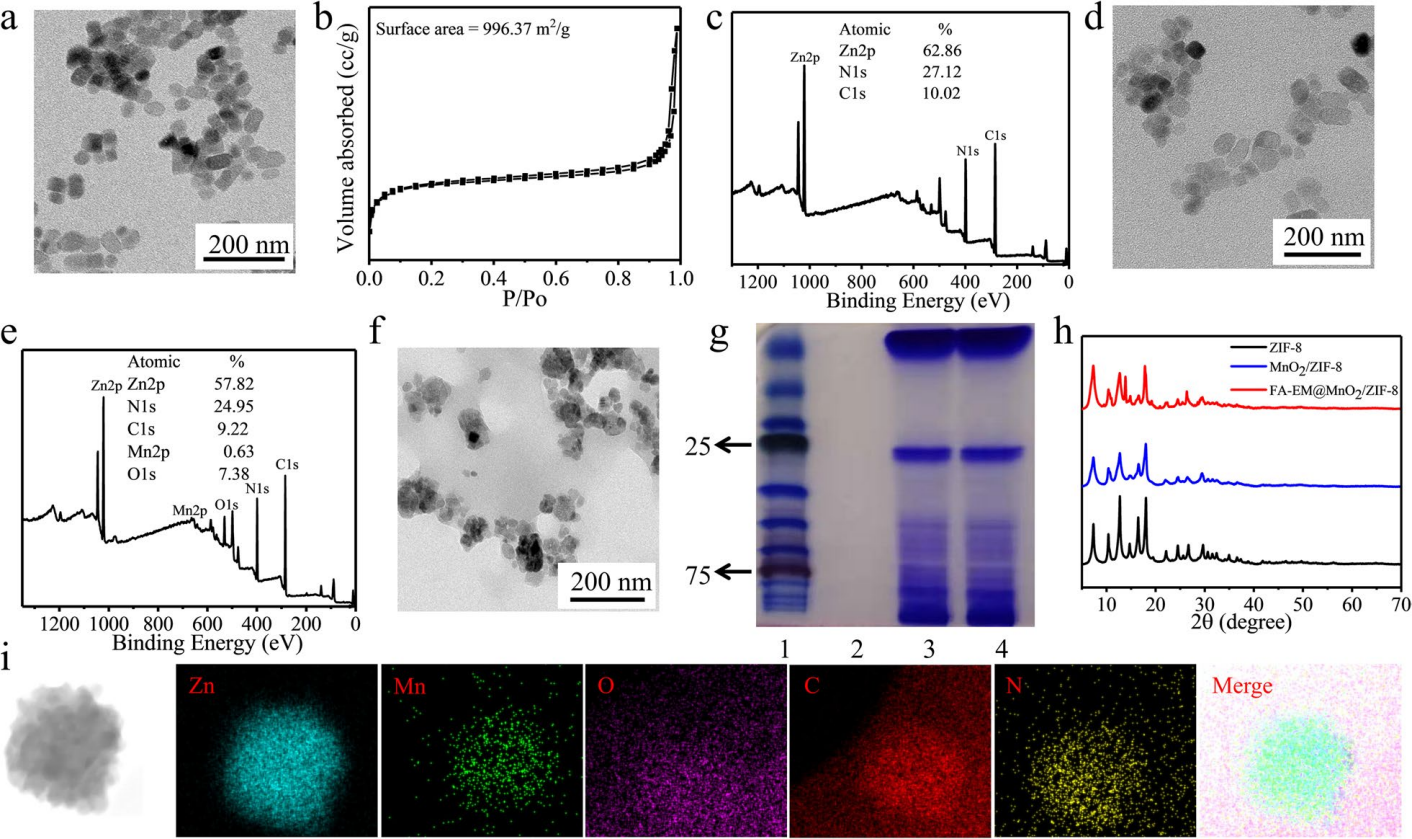
Characterization of FA-EM@MnO_2_/ZIF-8 nano-platform. **a** TEM, **b** BET, and **c** XPS of ZIF-8 nano-platform. **d** TEM and **e** XPS of MnO_2_/ZIF-8 nano-platform. **f** TEM of FA-EM@MnO_2_/ZIF-8 nano-platform. **g** Protein analysis of protein marker (1), MnO_2_/ZIF-8 (2), EM (3), and FA-EM@MnO_2_/ZIF-8 (4) by using SDS–PAGE. Samples were stained with Coomassie Brilliant. **h** XRD spectra of ZIF-8, MnO_2_/ZIF-8, and FA-EM@MnO_2_/ZIF-8 nano-platform. **i** High-angle annular dark-field scanning TEM (HAADF-STEM) image and element mapping of FA-EM@MnO_2_/ZIF-8 nano-platform

By biomimetic mineralization and coprecipitation, 2-ICA and Zn^2+^ were directly mixed and ZIF-8/ICG nano-platform was synthesized in one step. We found that the size of ZIF-8 nano-platform is about 60 nm, and the surface area is about 996.37 m^2^/g, which indicated that ZIF-8 nano-platform is ideal for drugs loading in tumor therapy (Fig. 1a and b). XPS results show that ZIF-8 nano-platform was mainly composed with zinc, nitrogen, and carbon, their percentages are about 68.86%, 27.12%, and 10.02%, respectively (Fig. 1c). After the surface of ZIF-8 as modified with MnO_2_, the size and the morphology of MnO_2_/ZIF-8 were not significantly changed (Fig. 1d and e). However, XPS results show that MnO_2_/ZIF-8 is mainly composed of zinc, nitrogen, carbon, oxygen, and manganese, they are uniformly dispersed in the nano-platform, with mass percentages of 57.85%, 24.94%, 9.22%, 7.38%, and 0.63%, respectively (Fig. 1e and i). In order to improve the biocompatibility and targeting of MnO_2_/ZIF-8, FA-EM was modified on the surface of MnO_2_/ZIF-8. TEM showed that the size of FA-EM@MnO_2_/ZIF-8 became larger, but the obtained FA-EM@MnO_2_/ZIF-8 nano-platform has a core–shell structure (Fig. 1f). Gel electrophoresis results showed that the protein composition of FA-EM@MnO_2_/ZIF-8 nano-platform kept well with that of pure EM, which indicated that the membrane protein of FA-EM fused to the surface of MnO_2_/ZIF-8 nano-platform was well preserved (Fig. 1g). From Fig. 1h, power X-ray diffraction (XRD) diagram shows that all the characteristic peaks of two samples (MnO_2_/ZIF-8, and FA-EM@MnO_2_/ZIF-8) are very similar to those of pure ZIF-8. The above results show that we successfully synthesized FA-EM@MnO_2_/ZIF-8 nano-platform.

**Fig. 2:**
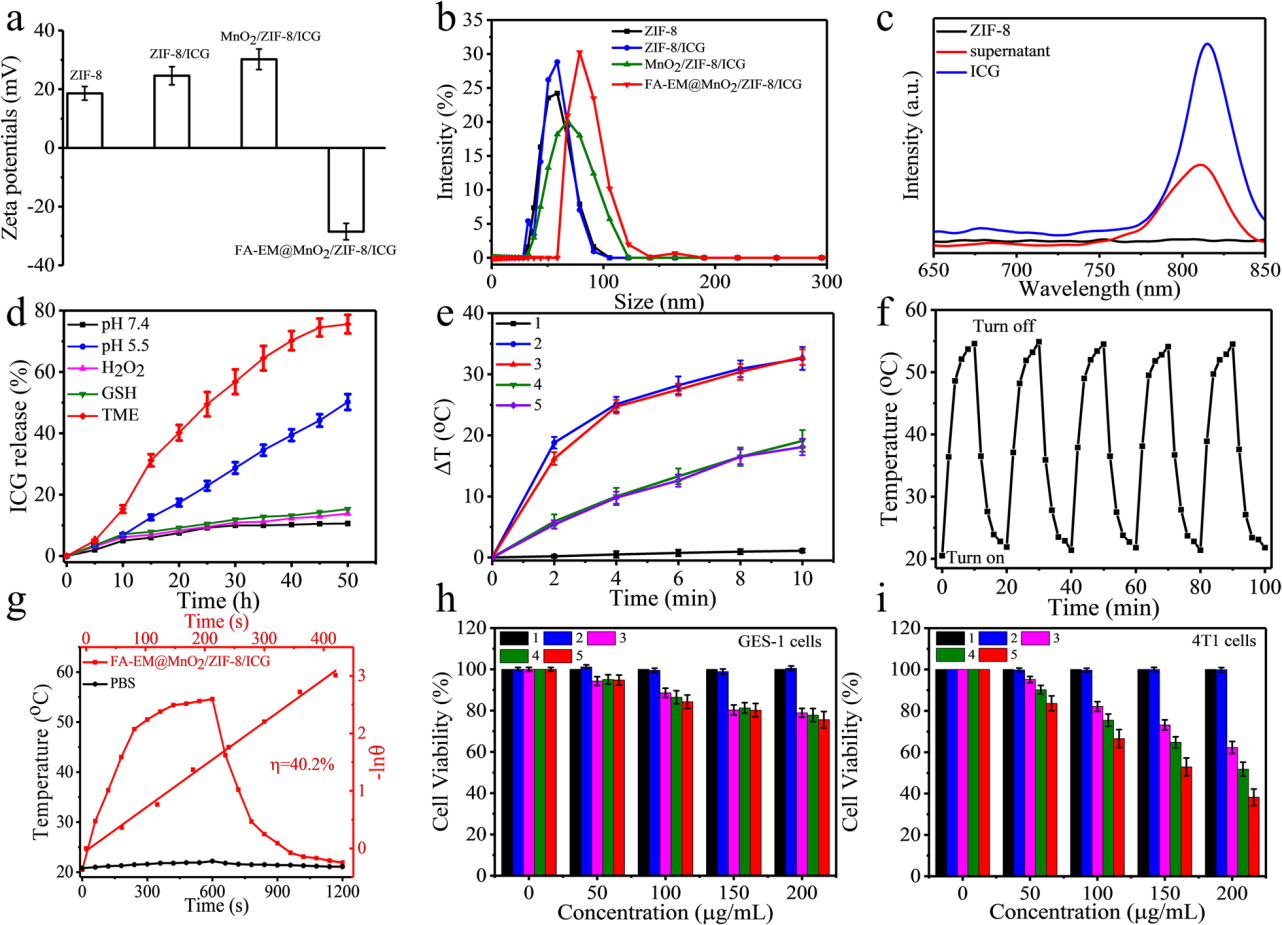
**a** Zeta potential of ZIF-8, ZIF-8/ICG, MnO_2_/ZIF-8/ICG, and FA-EM@MnO_2_/ZIF-8/ICG. **b** Size distribution of ZIF-8, ZIF-8/ICG, MnO_2_/ZIF-8/ICG, and FA-EM@MnO_2_/ZIF-8/ICG. **c** Fluorescence spectra of ZIF-8, the ICG solution before loading, and the residual ICG in the supernatant after loading. **d** In vitro profiles of ICG release from FA-EM@MnO_2_/ZIF-8/ICG nano-platform under conditions of pH 7.4, pH 5.5, H_2_O_2_, GSH and simulated TME. **e** Temperature changes of PBS (curve 1), 200 μg/mL ZIF-8/ICG (curve 2), 200 μg/mL FA-EM@MnO_2_/ZIF-8/ICG (curve 3), and 150 μg/mL FA-EM@MnO_2_/ZIF-8/ICG (curve 4) irradiated with 808 nm irradiation (2.0 W/cm^2^) for 10 min, and 200 μg/mL FA-EM@MnO_2_/ZIF-8/ICG (curve 5) irradiated with 808 nm irradiation (1.5 W/cm^2^) for 10 min. **f** Temperature changes of 200 μg/mL FA-EM@MnO_2_/ZIF-8/ICG disperse solution irradiation with 808 nm irradiation (2.0 W/cm^2^) for five turn on/off cycles. **g** Photothermal response of FA-EM@MnO_2_/ZIF-8/ICG solution and H_2_O treated with an NIR irradiation (808 nm, 1.5 W/cm^2^) for 600 s and then the irradiation was turned off. Viability of GES-1 cells (**h**) and 4T1cells (**i**) upon treated with PBS (1), PBS + NIR (2), ZIF-8/ICG + NIR (3), MnO_2_/ZIF-8/ICG + NIR (4), and FA-EM@MnO_2_/ZIF-8/ICG + NIR (5)

The ZIF-8/ICG nano-platform was prepared via direct self-assembly of 2-ICA and Zn^2+^ in the presence of ICG. Thus, zeta potential measurement was utilized to analyze the products formed at any synthesis stages (Fig. 2a). Compared with the data of pure ZIF-8, the zeta potential of ZIF-8/ICG nano-platform increased slightly, which indicated that ICG molecule was successfully incorporated into ZIF-8 crystals. The zeta potential of MnO_2_/ZIF-8/ICG nano-platform also increased when MnO_2_ was successfully modified on the surface of ZIF-8, but when FA-EM was modified on the surface of MnO_2_/ZIF-8/ICG nano-platform, the positive zeta potential of MnO_2_/ZIF-8/ICG became negative. In addition, the hydrodynamic diameter measurements were made to study the products formed at different synthesis stages (Fig. 2b). The size of FA-EM@MnO_2_/ZIF-8/ICG nano-platform is consistent with the measurement results of transmission electron microscope.

High drugs loading capacity and tumor tissues on-demand releasing are the key factor to enhance therapeutic effect and avoid unnecessary side effects. ZIF-8 was expected to be an excellent nano-platform for drugs loading and delivery because of its huge surface area. Figure 2c shows that the loading efficiency of ZIF-8 for ICG, a typical photosensitizer for tumor photothermal therapy with green fluorescence, reached for about 60% (ZIF-8/ICG), as determined by the fluorescence signal of ICG. The zeta potential also confirmed the successful loading of ICG. In vitro release behavior of ICG from FA-EM@MnO_2_/ZIF-8/ICG was evaluated in simulated physiological environment (pH = 7.4), pH 5.5, 30 μM H_2_O_2_, 10 mM GSH and TME-simulating environment (pH = 5.5, 10 mM GSH, 30 μM H_2_O_2_). As shown in Fig. 2d, under physiological conditions (pH = 7.4), only about 10% ICG was released after 50 h. The release behavior of ICG in GSH and H_2_O_2_ solution was similar. Nevertheless, 50.2% ICG was released in acidic solution (pH = 5.5), because ZIF-8 is prone to decompose under acidic environment, which can make ZIF-8 structure collapse leading ICG releasing [37]. Notably, in TME-simulating environment, more than 40% of ICG was released after 24 h, and about 75% of ICG was released after 50 h. In order to obtain more details of the reaction process, XPS analysis was carried out. As figure shows, after FA-EM@MnO_2_/ZIF-8/ICG nano-platform was immersed into the TME-simulating environment, H_2_O_2_ is accumulated on the EM surface, which promoted the formation of pores on the EM surface and promoted the solution reacted with MnO_2_ surface [38] and Mn^3+^ was converted into Mn^2+^, which was ascribed to the redox reaction between Mn^3+^ within the MnO_2_ and GSH (Additional file 1: Fig. S1a–b), leading MnO_2_ shell decomposed and promoted TEM-simulating solution entered into the core sites to promote ICG release [39, 40]. Thus, FA-EM@MnO_2_/ZIF-8/ICG could be used as an ideal platform for tumor treatment.

It is worth noting that ICG is the typical PS, and it has excellent photothermal conversion efficiency when exposed to NIR [41]. Therefore, the photothermal conversion effects of FA-EM@MnO_2_/ZIF-8/ICG were also evaluated via irradiation with NIR, showing super penetration into normal tissues, and the damage was negligible. Then, FA-EM@MnO_2_/ZIF-8/ICG dispersion solution with different concentrations (ICG concentration: 150 and 200 μg/mL) was irradiated at NIR with the power of 1.5 or 2.0 W/cm^2^. The photothermal conversion shows obvious dependence on time, concentration and radiation power. When the temperature increased for about 32.8 °C at the concentration of 200 μg/mL with the power of 2.0 W/cm^2^ for 10 min, the photothermal conversion efficiency (ŋ) for about 40.1%, which was suitable for tumor therapy than traditional photothermal agents (Fig. 2g) [42]. It should be noted that MnO_2_/ZIF-8/ICG and FA-EM@MnO_2_/ZIF-8/ICG nano-platform displayed similar temperature changes under the same conditions, which indicated that FA-EM camouflaged had no obvious influence of the irradiation absorption of MnO_2_/ZIF-8/ICG (Fig. 2e). In addition, FA-EM@MnO_2_/ZIF-8/ICG dispersion shown outstanding photothermal stability after treated with five turn on/off cycles, indicative of excellent reversible temperature changes (Fig. 2f). Therefore, the obtained FA-EM@MnO_2_/ZIF-8/ICG nano-platform shown enormous potential as a photothermal agent for anti-tumor therapy.

In the presence of NIR radiation, the cytotoxicity of ZIF-8/ICG nano-platform with different preparations of different kinds of cells was tested in vitro. For GES-1 cells, no significant toxicity of ZIF-8/ICG, MnO_2_/ZIF-8/ICG, and FA-EM@MnO_2_/ZIF-8/ICG was detected until the equivalent ICG dose reached 200 μg/mL (Fig. 2h), after 10 min of near-infrared irradiation, which was also the same dose selected for the in vitro analysis of 4T1 cells. 4T1 cells were incubated for 3 h with each nano-platform before being irradiated for 10 min. Comparing with GES-1 cells viability, the 4T1 cells killing efficiency of each nano-platform exhibited ICG dose-dependent cytotoxicity in the presence of NIR irradiation, and only about 38.2% 4T1 cells viability under NIR irradiation with the power of 2.0 W/cm^2^ at the dose of FA-FA-EM@MnO_2_/ZIF-8/ICG for 200 μg/mL (Fig. 2i). Flow-cytometry apoptosis assay also verified that the cytotoxicity of FA-EM@MnO_2_/ZIF-8/ICG nano-platform to 4T1 cells under near-infrared radiation was higher than that of GES-1 cells (Additional file 1: Fig. S2a–b). Therefore, FA-EM@MnO_2_/ZIF-8/ICG can effectively kill cancer cells as a nano-platform, and avoid unnecessary side effects to normal cells.

**Fig. 3:**
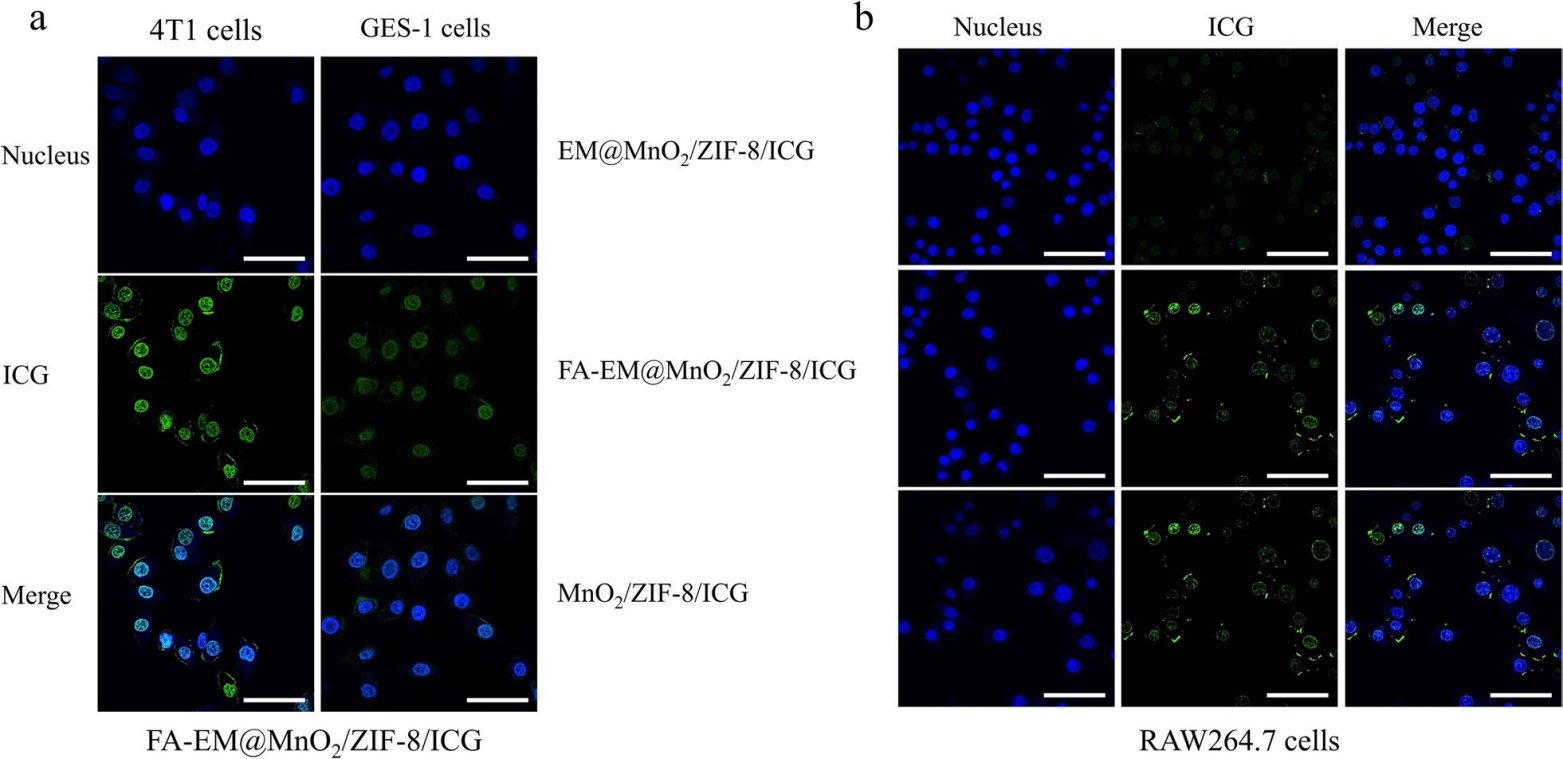
**a** CLSM images of 4T1 and GES-1 cells incubated with FA-EM@MnO_2_/ZIF-8/ICG nano-platform for 3 h. **b** CLSM images of macrophage cells incubated with EM@MnO_2_/ZIF-8/ICG, FA-EM@MnO_2_/ZIF-8/ICG, and MnO_2_/ZIF-8/ICG nano-platform for 3 h. Images show cell nuclei stained by DAPI (blue), ICG fluorescence in cells (green), and the merged overlap of the two images. Scale bars: 30 μm

To gain insight into the details of FA-EM@MnO_2_/ZIF-8/ICG nano-platform has high cytotoxicity to cancer cells than normal cells upon being exposed under NIR irradiation. Flow-cytometry analysis showed that the FA-EM@MnO_2_/ZIF-8/ICG nano-platform was swallowed up by 4T1 cells more than GES-1 cells (Additional file 1: Fig. S3a–b). The fluorescence intensity of ICG in 4T1 cells was approximately fourfold stronger than that of normal GES-1 cells (Fig. 3a). This is because FA-EM@MnO_2_/ZIF-8/ICG nano-platform can specially bind with folate receptor (FA) which is overexpressed on the surface of 4T1 cells, resulting in more FA-EM@MnO_2_/ZIF-8/ICG nano-platform phagocytosis by 4T1 cells. It has been reported in many literatures that nanoparticles coating erythrocyte membrane (EM) can give bionic nanoparticles longer blood circulation and avoid being cleared by the immune system [47, 48]. Therefore, we further studied the electromagnetic masking effect and evaluated the uptake behavior of macrophages on different nano-platforms by confocal laser scanning microscope. Figure 3b shows that compared with MnO_2_/ZIF-8/ICG nano-platform, the binding or internalization of macrophages to EM@MnO_2_/ZIF-8/ICG nano-platform is significantly reduced. The results of flow cytometry also confirmed that once FA-EM was transferred to the surface of MnO_2_/ZIF-8/ICG, the FA-EM@MnO_2_/ZIF-8/ICG nano-platform can be effectively prevented from being destroyed by macrophages (Additional file 1: Fig. S4a-c). This was consistent with previous studies, which means that EM clock can reduce the clearance of nanomaterials by the reticuloendothelial systems, and it is proved that EM camouflage can increase the cycle time of ZIF-8 nano-platform in vivo [43]. It should be pointed out that the fluorescence intensity of FA-EM@MnO_2_/ZIF-8/ICG nano-platform is slightly stronger than that of EM@MnO_2_/ZIF-8/ICG nano-platform, which indicates that the stealth effects of EM are the least damaged after FA is modified to EM surface. Above results indicate that FA-EM@MnO_2_/ZIF-8/ICG nano-platform has the ability of targeted delivery and invisibility in blood circulation, which can enhance tumor treatment effects.

**Fig. 4:**
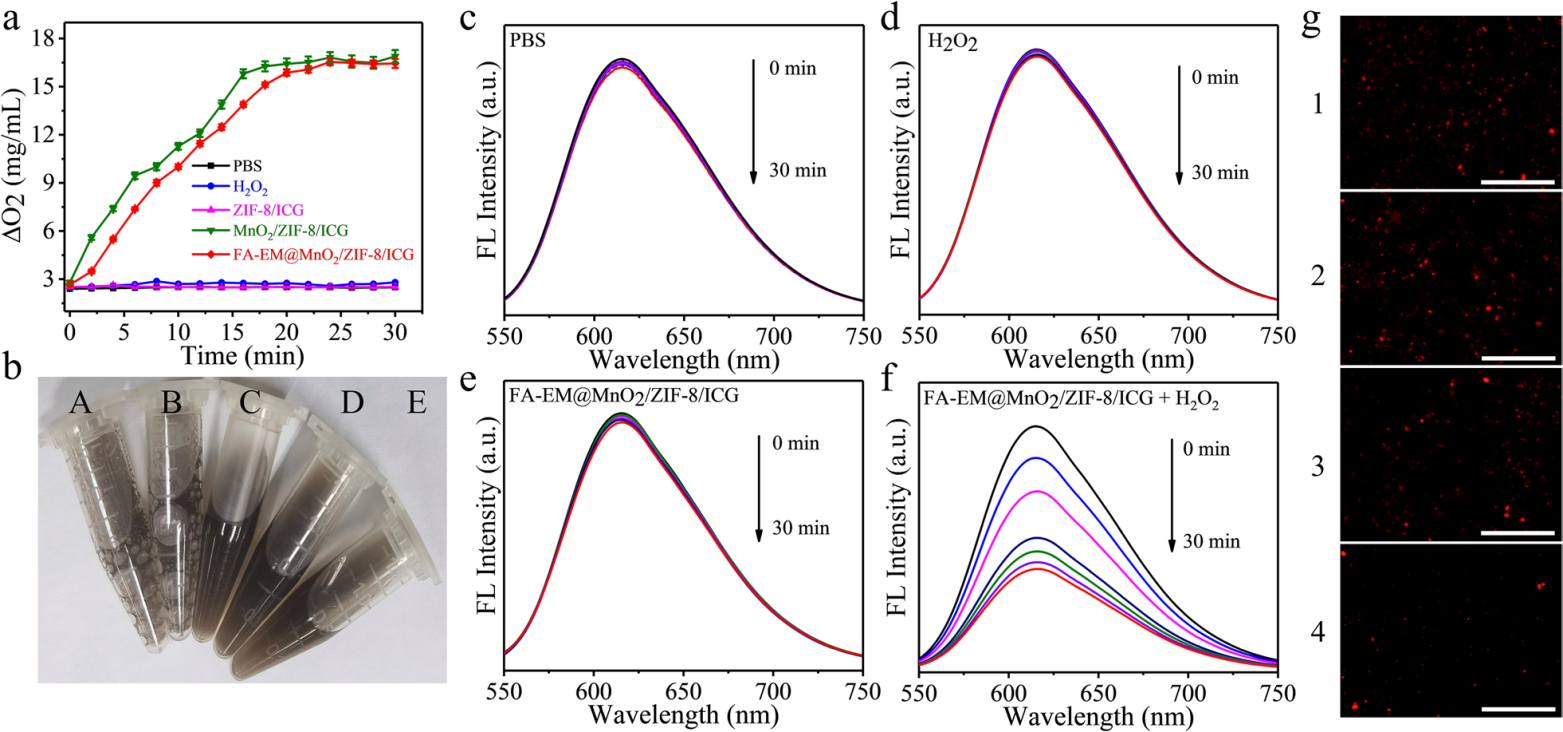
**a** H_2_O_2_-triggered O_2_ generation in different solutions. **b** Photograph of H_2_O_2_-triggered O_2_ generation in different solutions. (A: MnO_2_/ZIF-8/ICG in H_2_O_2_ solution; B: FA-EM@MnO_2_/ZIF-8/ICG in H_2_O_2_ solution; C: ZIF-8/ICG in H_2_O_2_ solution; D: MnO_2_/ZIF-8/ICG in PBS solution; E: FA-EM@MnO_2_/ZIF-8/ICG in PBS solution). **c** Fluorescence spectra of RDPP in PBS (**c**); H_2_O_2_ (**d**); FA-EM@MnO_2_/ZIF-8/ICG (**e**); and FA-EM@MnO_2_/ZIF-8/ICG + H_2_O_2_ (**f**). **g** CLSM images of RDPP in 4T1 cells without any treatments (1); and treated with H_2_O_2_ (2); FA-EM@MnO_2_/ZIF-8/ICG (3); and FA-EM@MnO_2_/ZIF-8/ICG + H_2_O_2_ (4). Scale bars: 300 μm

Weak acidity, hypoxia, increased H_2_O_2_ levels, and high concentration of GSH are the typical characteristics of the TME [44, 45]. It is reported that MnO_2_ has catalase-like activity which can trigger O_2_ generation from H_2_O_2_ [46]. In order to study the O_2_ generation capacity of FA-EM@MnO_2_/ZIF-8/ICG nano-platform in H_2_O_2_ environment, more O_2_ could be detected upon MnO_2_/ZIF-8/ICG and FA-EM@MnO_2_/ZIF-8/ICG nano-platform immersed into the H_2_O_2_ solution compared with ZIF-8/ICG immersed into H_2_O_2_ solution, MnO_2_/ZIF-8/ICG in PBS solution, and FA-EM@MnO_2_/ZIF-8/ICG in PBS solution by a dissolved oxygen meter (Fig. 4a). In addition, when MnO_2_/ZIF-8/ICG and FA-EM@MnO_2_/ZIF-8/ICG nano-platforms were immersed in H_2_O_2_ solution, more bubbles could be detected, but when ZIF-8/ICG nano-platforms were immersed into H_2_O_2_ solution, no bubbles were generated (Fig. 4b). The above results indicated that MnO_2_ has the activity similar to catalase. It should be noted that FA-EM@MnO_2_/ZIF-8/ICG nano-platform can’t produce O_2_ rapidly at the begin time than MnO_2_/ZIF-8/ICG, which would ascribe to the H_2_O_2_ solution infiltrated through the FA-EM and entered into the core area of FA-EM@MnO_2_/ZIF-8/ICG to react with MnO_2_, but FA-EM cloaked can’t effect FA-EM@MnO_2_/ZIF-8/ICG catalase-like activity. In addition, RDPP as the typical oxygen-sensitive probe, whose fluorescence can be quenched in O_2_ environment [47]. As expected, the fluorescence intensity of RDPP was significantly decreased upon FA-EM@MnO_2_/ZIF-8/ICG nano-platform immersed into the H_2_O_2_ solution with time gone, which is indicative that O_2_ was gradually generated in the presence of both H_2_O_2_ and FA-EM@MnO_2_/ZIF-8/ICG nano-platform (Fig. 4f). As the control, O_2_ can hardly be detected in the solution only containing PBS, H_2_O_2_, and FA-EM@MnO_2_/ZIF-8/ICG dispersion solution (Fig. 4c–e). Besides, intracellular fluorescence signal of RDPP was further tested through 4T1 cells incubated with FA-EM@MnO_2_/ZIF-8/ICG. From Fig. 4g shown, the fluorescence intensity of RDPP was quenched upon 4T1 cells incubated with FA-EM@MnO_2_/ZIF-8/ICG nano-platform, and the fluorescence intensity was almost quenched once H_2_O_2_ added. In contrast, 4T1cells without FA-EM@MnO_2_/ZIF-8/ICG nano-platform did not show any obviously fluorescence quenched (Fig. 4g). The above results clearly indicated that MnO_2_ in FA-EM@MnO_2_/ZIF-8/ICG can catalyze the H_2_O_2_-triggered intracellular O_2_ production, so as to overcome the hypoxia TME and provide more O_2_ for subsequent photodynamic therapy.

**Fig. 5:**
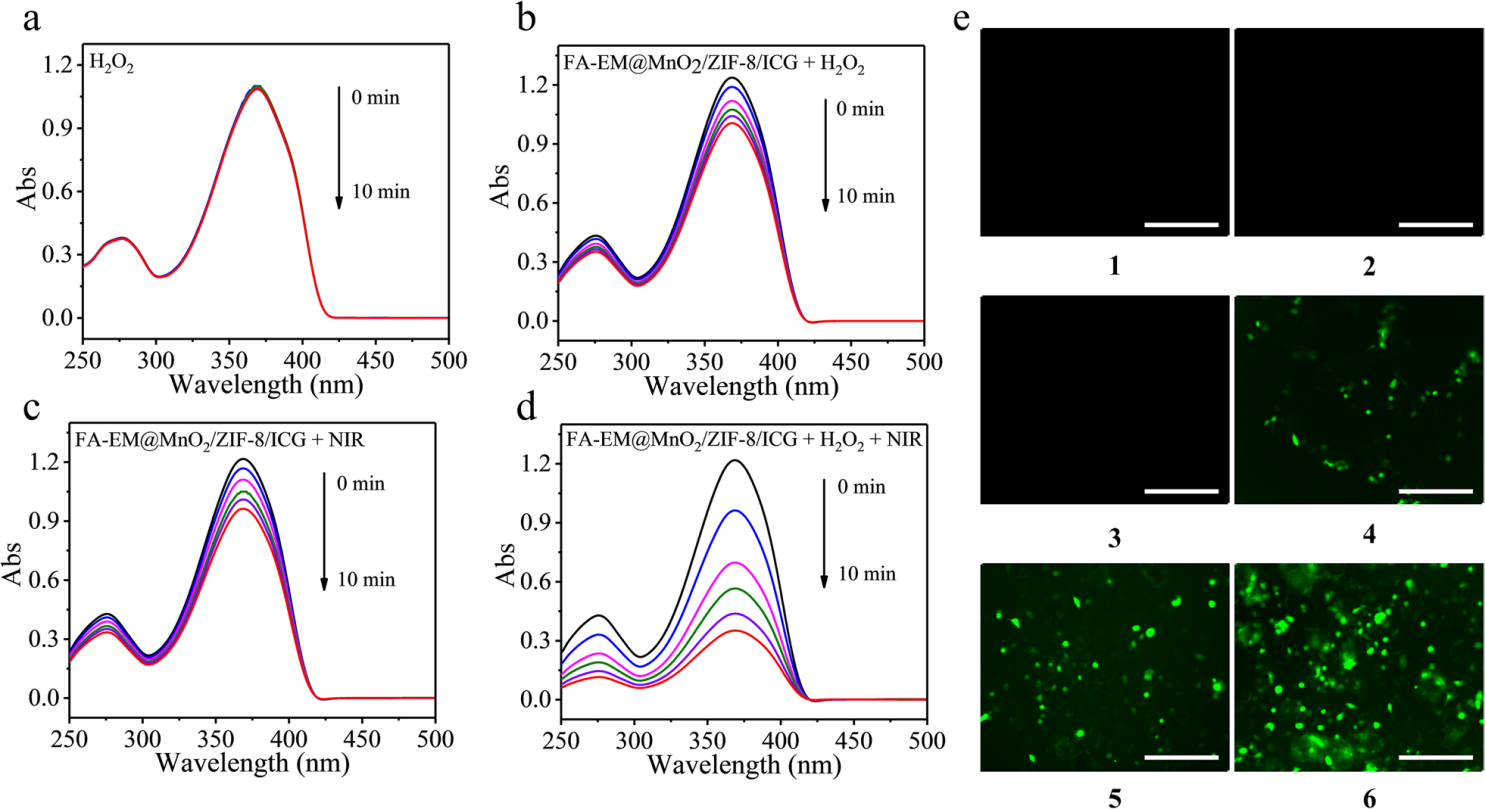
UV–vis absorption of DPBF in H_2_O_2_ (**a**), FA-EM@MnO_2_/ZIF-8/ICG + H_2_O_2_ (**b**), FA-EM@MnO_2_/ZIF-8/ICG + NIR (**c**) and FA-EM@MnO_2_/ZIF-8/ICG + H_2_O_2_ + NIR (**d**) at different time points. **e** CLSM images of DCF in 4T1 cells without any treatments (1), and treated with NIR irradiation only (2), H_2_O_2_ (3), FA-EM@MnO_2_/ZIF-8/ICG + H_2_O_2_ (4), FA-EM@MnO_2_/ZIF-8/ICG + NIR (5), and FA-EM@MnO_2_/ZIF-8/ICG + H_2_O_2_ + NIR (6). Scale bars: 300 μm

In order to investigate whether reactive oxygen (ROS), such as hydroxyl radical (^.^OH), and singlet oxygen (^1^O_2_) could be produced in the presence of FA-EM@MnO_2_/ZIF-8/ICG at TME with or without NIR irradiation. As a probe of extracellular ROS, DPBF can be oxidized by ROS, and its UV–vis absorption decreases [48]. According to Fig. 5a–d, the UV–Vis absorption values of DPBF in different environments have decreased. Obviously, the UV–visible absorption value of DPBF in FA-EM@MnO_2_/ZIF-8/ICG + H_2_O_2_ + NIR irradiation group decreased significantly, which indicates that the ROS was the highest. In addition, DCFH-DA was used as an indicator to detect the production of intracellular ROS through different treatments. DCFH-DA can be oxidized by intracellular ROS into stronger green fluorescent DCF [49]. By contrast with PBS group (1), treated with NIR irradiation only group (2), and H_2_O_2_ group (3), a green fluorescence signal was clearly presented after 4T1 cells treated with FA-EM@MnO_2_/ZIF-8/ICG or FA-EM@MnO_2_/ZIF-8/ICG + NIR. Moreover, the green fluorescence signal became stronger with H_2_O_2_ added, which was ascribed to FA-EM@MnO_2_/ZIF-8/ICG was beneficial to trigger the catalytic activity to produce more O_2_ and OH, and then improve the PDT process under NIR irradiation (Fig. 4e). These results correspond with the DPBF results, confirming that the obtained FA-EM@MnO_2_/ZIF-8/ICG nano-platform has the catalase-like activity can produce more O_2_ and ROS to overcome hypoxia TME with the existence of H_2_O_2_ and improve PDT efficacy.

**Fig. 6:**
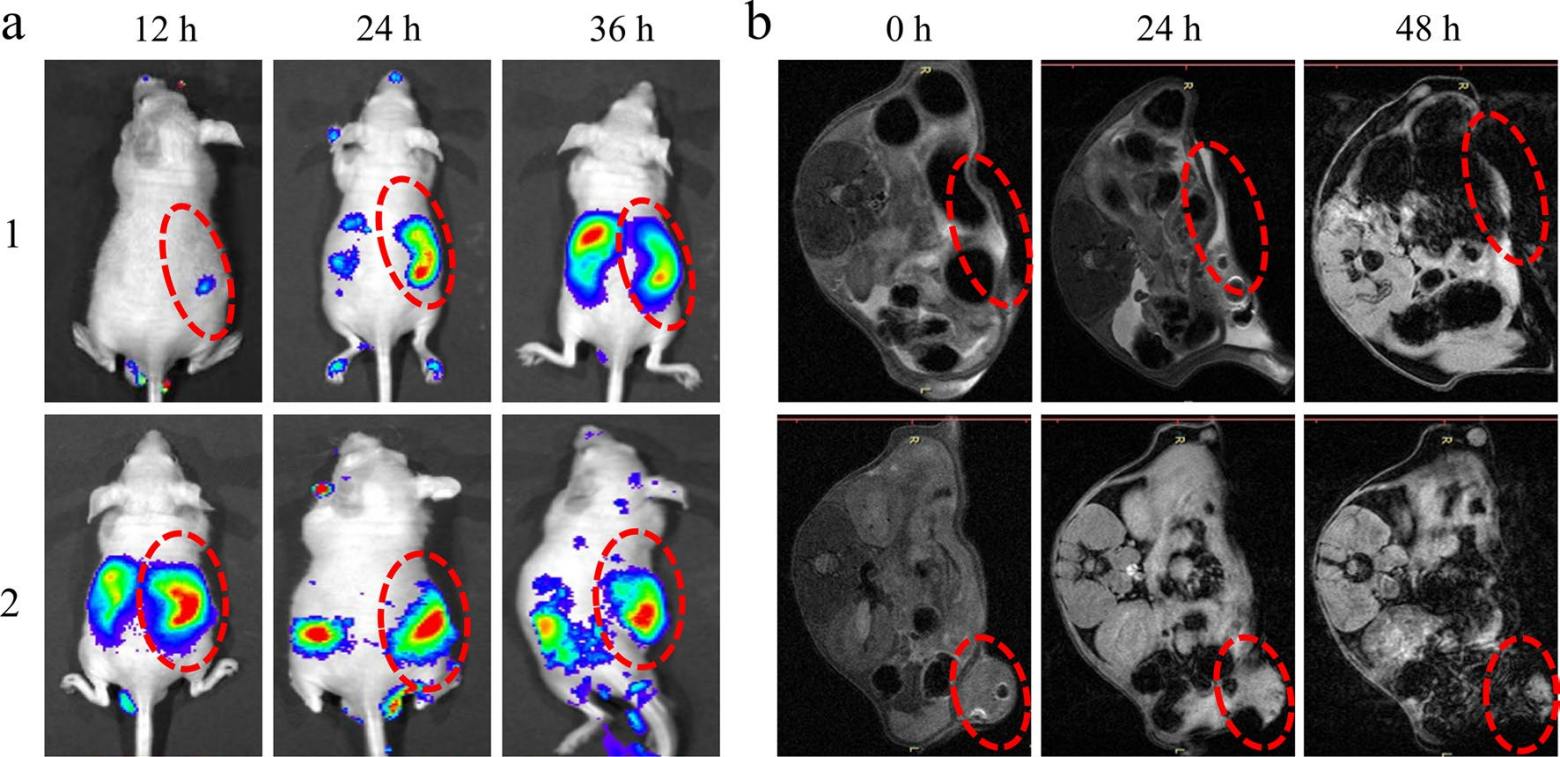
**a** In vivo luminescence imaging and **b** MRI of 4T1-bearing mice at designed time after treated with MnO_2_/ZIF-8/ICG (1) and FA-EM@MnO_2_/ZIF-8/ICG (2) nano-platform. The red dashed line represents tumor tissue

The excellent stimulation response behavior and anti-tumor effects of FA-EM@MnO_2_/ZIF-8/ICG forced us to further evaluate its anti-tumor activity in vitro. In order to explore the appropriate time for tumor treatment, the in vivo distribution of FA-EM@MnO_2_/ZIF-8/ICG nano-platform at the designed time was analyzed by luminescence imaging and magnetic resonance imaging (MRI). Comparing with MnO_2_/ZIF-8/ICG nano-platform, after FA-EM@MnO_2_/ZIF-8/ICG was injected into the 4T1 xenograft tumor mouse model, more ICG fluorescence signals were observed in the tumor area, and the fluorescence signal gradually weakened after 24 h of injection (Fig. 6a). The MRI results also confirmed that more FA-EM@MnO_2_/ZIF-8/ICG nano-platforms accumulated at the tumor areas 24 h after the injection, which indicated that FA-EM@MnO_2_/ZIF-8/ICG nano-platforms could achieve the tumor-targeted delivery of ICG through the folate receptors overexpressed on the tumor cell surface (Fig. 6b). Therefore, above results indicate that FA-EM@MnO_2_/ZIF-8/ICG nano-platform could be effectively delivered to the tumor areas 24 h after injection, and the nonspecific uptake by other organs can be avoided during the transport process.

**Fig. 7:**
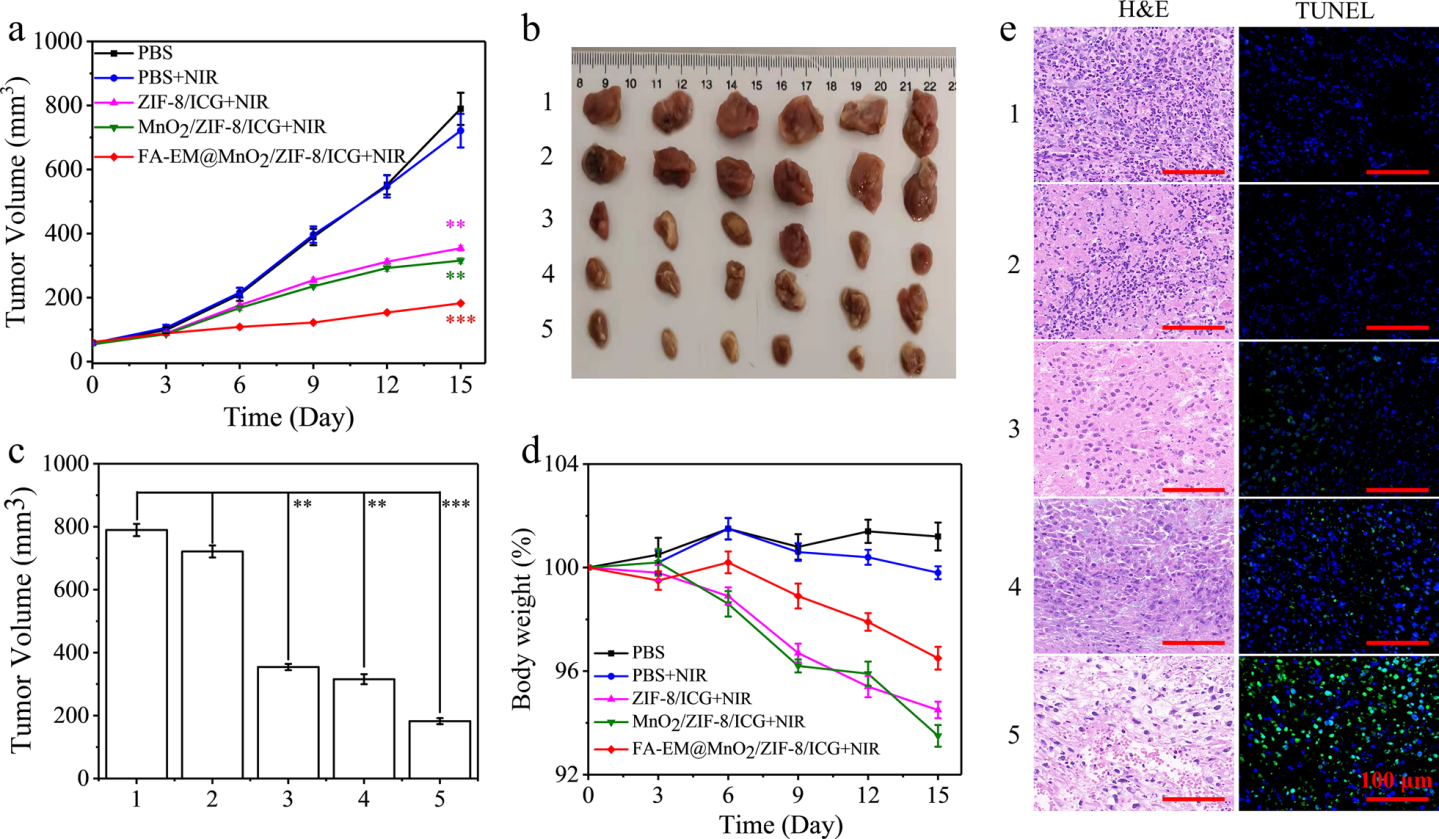
**a** The tumor volumes in six different groups after treatment. **b** Photographs of the tumors on day 15 post-injection, and **c** Mean tumor weights on day 15 after the last treatment of PBS (1), PBS + NIR (2), ZIF-8/ICG + NIR (3), MnO_2_/ZIF-8/ICG + NIR (4), FA-EM@MnO_2_/ZIF-8/ICG + NIR (5). **d** Body weight of mice in different groups during treatment. **e** H&E (left) and TUNEL staining (right) of tumor slices collected from 4T1 tumor-bearing mice after treatment of PBS (1), PBS + NIR (2), ZIF-8/ICG + NIR (3), MnO_2_/ZIF-8/ICG + NIR (4), FA-EM@MnO_2_/ZIF-8/ICG + NIR (5). Scale bars: 100 μm. Significant differences (*: *p* < 0.05; **: *p* < 0.01; ***: *p* < 0.001) among different groups are shown

Therefore, we established a 4T1 xenograft tumor model to detect the anti-tumor effects of FA-EM@MnO_2_/ZIF-8/ICG nano-platform under NIR irradiation. NIR irradiation was performed with the power of 2.0 W/cm^2^ every 3 days after 24 h post-injection. As shown in Fig. 7a–c, the tumor volume of different nano-platforms (ZIF-8/ICG, MnO_2_/ZIF-8/ICG, and FA-EM@MnO_2_/ZIF-8/ICG) after treatment showed some degree of inhibition. Especially, when FA-EM@MnO_2_/ZIF-8/ICG nano-platform treated mice were exposed under near-infrared irradiation, the tumor volume decreased significantly, which was attributed to the tumor-targeted delivery behavior of FA and the immune escape ability of EM on the surface of FA-EM@MnO_2_/ZIF-8/ICG nano-platform. Once in TEM, high levels of H_2_O_2_ can trigger the production of O_2_ to improve ameliorate hypoxia TME and provide more ROS to kill cancer cells under NIR irradiation. What’s more, compared with PBS, PBS + NIR, and pure NIR irradiation group, the weight of mice treated with different nano-platform did not show significant changes, and the overall mental state of each mouse remained good (Fig. 7d and Additional file 1: Fig. S5a). In order to further evaluate the anti-tumor effect of each nano-platform, H&E and TUNEL staining were used to evaluate the apoptosis and necrosis of cancer cells in tumor tissues (Fig. 7e and Additional file 1: Fig. S5b). Compared with PBS PBS + NIR, and pure NIR irradiation group, abnormal areas were detected in the treatment groups of ZIF-8/ICG + NIR, MnO_2_/ZIF-8/ICG + NIR, and FA-EM@MnO_2_/ZIF-8/ICG + NIR. Especially in FA-EM@MnO_2_/ZIF-8/ICG + NIR group, more nuclear pyknosis, nuclear lysis, and vacuolation were detected in the tumor tissues, which indicated a more significant killing effect on cancer cells. In addition, more green fluorescence detected by TUNEL also confirmed that FA-EM@MnO_2_/ZIF-8/ICG + NIR treatment can effectively inhibit the proliferation and induce the apoptosis of cancer cells compared with other treatments. These results indicate that once FA-EM@MnO_2_/ZIF-8/ICG nano-platform is exposed to near-infrared radiation, it can effectively inhibit the growth of tumor and reduce unnecessary side effects.

In order to investigate the biocompatibility of FA-EM@MnO_2_/ZIF-8/ICG nano-platform to main organs (heart, liver, spleen, lung, and kidney), the changes of main organs after 15 d of treatment were detected by H&E staining. It can be seen from Fig. 7 and Additional file 1: Fig. S6 that no obvious toxicity was detected in FA-EM@MnO_2_/ZIF-8/ICG group; There was no significant change in H&E staining of tissue sections of different organs. These results indicated that FA-EM@MnO_2_/ZIF-8/ICG nano-platform can be used as a safe and effective nano-platform for tumor treatment.

## Conclusion

In summary, we have developed an O_2_-generating FA-EM-cloaked ZIF-8 based photosensitizer delivery system (FA-EM@MnO_2_/ZIF-8/ICG) for fluorescence-guiding PDT. The constructed FA-EM@MnO_2_/ZIF-8/ICG nano-platform has the following merits: (1) It avoids phototoxicity and fluorescence quenching effects of ICG during transportation process; (2) FA-EM@MnO_2_/ZIF-8/ICG can avoid eliminated by body immune system and target delivery to tumor tissues; (3) by decorated MnO_2_ on the surface of ZIF-8, the final FA-EM@MnO_2_/ZIF-8/ICG has the ability of self-supplied of O_2_ to enhance subsequently PDT. These results proved that FA-EM@MnO_2_/ZIF-8/ICG has shown excellent biocompatibility, self-sufficiency of O_2_ to overcome hypoxia TME further improving the photodynamic activity to therapy tumors, and environment-responsive drugs sustained released. In vitro and in vivo results illustrated that as-prepared FA-EM@MnO_2_/ZIF-8/ICG nano-platform exhibited excellent anti-tumor effects, which would offer comprehensive effects in tumor therapy and hold great promise for clinical practice.

## Supplementary Information

**Additional file 1.** **Fig. S1.** Narrow XPS scan spectra of Mn2p in FA-EM@MnO2/ZIF-8/ICG (a) and FA-EM@MnO_2_/ZIF-8/ICG in TME-simulating solution (b). **Fig. S2.** Viability of GES-1 cells (a) and 4T1cells (b) upon treated with PBS (1), PBS + NIR (2), ZIF-8/ICG + NIR (3), MnO_2_/ZIF-8/ICG + NIR (4), and FA-EM@MnO_2_/ZIF-8/ICG + NIR (5) by flow-cytometry. **Fig. S3.** Flow-cytometry analyzed 4T1 and GES-1 cells uptake behavior for FA-EM@MnO_2_/ZIF-8/ICG nano-platform. **Fig. S4.** Flow-cytometry analyzed macrophage cells phagocytosis behavior with EM@MnO_2_/ZIF-8/ICG (a), FA-EM@MnO_2_/ZIF-8/ICG (b), and MnO_2_/ZIF-8/ICG (c) nano-platform. **Fig. S5.** (a) Photographs of the tumors on day 15 post-injection after the last treatment of pure NIR irradiation. (b) H&E (left) and TUNEL staining (right) of tumor slices collected from 4T1 tumor-bearing mice after treatment of pure NIR irradiation. Scale bars: 100 μm. (c) H&E staining of various organs collected from 4T1 tumor-bearing mice after treatments of pure NIR irradiation. Scale bars: 200 μm. **Fig. S6.** H&E staining of various organs collected from 4T1 tumor-bearing mice after different treatments of PBS (1), PBS + NIR (2), ZIF-8/ICG + NIR (3), MnO_2_/ZIF-8/ICG + NIR (4), FA-EM@MnO_2_/ZIF-8/ICG + NIR (5). Scale bars: 150 μm.

## Data Availability

The datasets generated during and analyzed during the current study are available from the corresponding authors on reasonable request.

## Abbreviations

ICG: Indole green
PDT: Photodynamics therapy
TME: Tumor microenvironment
ROS: Reactive oxygen
NIR: Near infrared
RDPP: [Ru(dpp)_3_]Cl_2_
DPBF: 1, 3-Diphenylisobenzofuran
DCFH-DA: 2′, 7′-Dichlorofluorescein diacetate
TEM: Transmission electron microscope
XRD: X-ray diffraction
MRI: Magnetic resonance imaging
CLSM: Confocal laser scanning microscopy
DSPE-PEG-FA: 1,2-Distearoyl-sn-glycero-3-phosphoethanolamine-*N*-[folate (polyethylene glycol)-2000]
DAPI: 4′,6-Diamidino-2-phenylindole
EM: Erythrocyte membrane
FA: Folic acid
