# Supplementary material for: H2O2/pH Dual-Responsive Biomimetic Nanoenzyme Drugs Delivery System for Enhanced Tumor Photodynamic Therapy
Source: Nanoscale Res Lett. 2022 Oct 29;17:103. doi: 10.1186/s11671-022-03738-9 (PMC9618007; doi:10.1186/s11671-022-03738-9)
Supplement: Supplementary file 1 — Additional file 1. Fig. S1. Narrow XPS scan spectra of Mn2p in FA-EM@MnO2/ZIF-8/ICG (a) and FA-EM@MnO2/ZIF-8/ICG in TME-simulating solution (b). Fig. S2. Viability of GES-1 cells (a) and 4T1cells (b) upon treated with PBS (1), PBS + NIR (2), ZIF-8/ICG + NIR (3), MnO2/ZIF-8/ICG + NIR (4), and FA-EM@MnO2/ZIF-8/ICG + NIR (5) by flow-cytometry. Fig. S3. Flow-cytometry analyzed 4T1 and GES-1 cells uptake behavior for FA-EM@MnO2/ZIF-8/ICG nano-platform. Fig. S4. Flow-cytometry analyzed macrophage cells phagocytosis behavior with EM@MnO2/ZIF-8/ICG (a), FA-EM@MnO2/ZIF-8/ICG (b), and MnO2/ZIF-8/ICG (c) nano-platform. Fig. S5. (a) Photographs of the tumors on day 15 post-injection after the last treatment of pure NIR irradiation. (b) H&E (left) and TUNEL staining (right) of tumor slices collected from 4T1 tumor-bearing mice after treatment of pure NIR irradiation. Scale bars: 100 μm. (c) H&E staining of various organs collected from 4T1 tumor-bearing mice after treatments of pure NIR irradiation. Scale bars: 200 μm. Fig. S6. H&E staining of various organs collected from 4T1 tumor-bearing mice after different treatments of PBS (1), PBS + NIR (2), ZIF-8/ICG + NIR (3), MnO2/ZIF-8/ICG + NIR (4), FA-EM@MnO2/ZIF-8/ICG + NIR (5). Scale bars: 150 μm. [file 11671_2022_3738_MOESM1_ESM.docx]

**Additional file**

**H_2_O_2_/pH dual-responsive biomimetic nanoenzyme drugs delivery system for enhanced tumor** **photodynamic therapy**

Xinyuan Li,^1^ Qing Ji,^3^ Chao Yan,^1^ Ziyu Zhu,^1^ Zhihui Yan,^1^ Ping Chen,^1^ Yisen Wang^4*^ and Li Song^2*^

1. The Affiliated Huai’an Hospital of Xuzhou Medical University and The Second People’s Hospital of Huai’an, No.62, Huaihai Road (S.), Huai’an 223002, China.
2. YanCheng NO.1 People’s Hospital, YanCheng 224001, China.
3. School of Medicine, Jiangsu University, Zhenjiang, 212013, China.
4. Institute of Translational Medicine, Medical College, Yangzhou University, Yangzhou 225009, China.

Corresponding: Yisen Wang

Email address: [wangyisen2023@163.com](mailto:wangyisen2023@163.com)

Mainly corresponding: Li Song

Email address:sl1035073700@163.com

Address: YanCheng NO.1 People’s Hospital, YanCheng 224001, China.

**Experimental section**

**Materials and Reagents**

KMnO_4_, hydrogen peroxide, 2-methylimidazole and Zn(NO_3_)_2_·6H_2_O purchased from Sinopharm Chemical Reagent (Beijing, China). Indocyanine green (ICG), 4’,6-diamidino-2-phenylindole (DAPI), glutathione (GSH, reduced), cell counting kit-8 (CCK-8), 1,3-diphenylisobenzofuran (DPBF), [Ru(dpp)_3_]Cl_2_ (RDPP), 2′,7′-dichlorofluorescein diacetate (DCFH-DA) and 1,2-distearoyl-sn-glycero-3-phosphoethanolamine-N-[folate (polyethylene glycol)-2000] (DSPE-PEG-FA) bought from Aladdin (Shanghai, China). Fetal bovine serum (FBS), dulbecco’s modified eagle medium (DMEM), phosphate buffer (PBS) and 1640 medium purchased from Gibco (Shanghai, China). All chemicals were used in this work without further purification.

**Apparatus and Procedures**

Transmission electron microscopy (TEM) images were obtained on a transmission electron microscope (JEOL 2100, Japan). X-ray powder diffraction (XRD) measurement, Brunauer-Emmett-Teller (BET) surface area, ζ-potential measurements and mapping were performed from on an X-ray diffractometer (GBC MMA Instrument), N_2_ adsorption-desorption isotherms (NovaWin 1000e, USA), and a NanoBrook Omni (Brookhaven, USA), respectively. Infrared thermal photos and temperature changes were recorded on the infrared thermal camera (HT-19, Guangzhou, China). 808 nm near-infrared irradiation was performed by a fiber-coupled NIR laser (MDL-N-808 nm-10W, Beijing Laserwave OptoElectronics Technology Co., Ltd., Beijing, China). Confocal laser scanning microscopy (CLSM, Nikon, Japan) was performed to detect cytophagocytic behavior.

**Experimental section**

**Synthesis of ZIF-8/ICG**

ZIF-8/ICG nano-platform was prepared according to previous methods.^1^ Briefly, Zn(NO_3_)_2_·6H_2_O (1470 mg) and ICG (100 mg) were added into the 10 mL PBS solution and stirring for 15 min to obtain homogeneous solution A. Then, 2-methylimidazole (810 mg) was also added into the 10 mL PBS stirring at 800 rpm for 15 min to obtain solution B. Finally, solution B was slowly dropped into the solution A, and then the mixed solution was continued stirring at 800 rpm for 15 min. The obtained ZIF-8/ICG was washed with PBS for three times, and the supernatant solution was kept for confirming the ICG content through high-performance liquid chromatography (HPLC). The drugs loading efficiency was calculated by the following equation: Drugs loading efficiency = (initial weight of drugs - weight of drugs in supernatant)/weight of drugs loaded nanoparticles.^2^

**Synthesis of MnO_2_/****ZIF-8/ICG**

10 mg ZIF-8/ICG was dispersed in 10 mL PBS solution and ultra-sonicated for 10 min to obtain homogeneous dispersion. Then, 10 mL aqueous solution of KMnO_4_ solution (1 mg/mL) was added dropwise and stirred at 800 rpm for 4 h. Then, the color of ZIF-8/ICG turned into brown after MnO_2_ successful coated into the surface of ZIF-8/ICG.^3^

**Cellular uptake**

First, RAW264.7 cells (mouse macrophage cells), 4T1 cells (mouse breast cancer cells) and GES-1 cells (human gastric mucosa cells) were respectively seeded into six-well plate and incubated for 24 h. Different formulation of nano-platform (100 μg/mL) were added into each well and incubated for another 3 h. Then, the cells were fixed with 4% paraformaldehyde and added DAPI for 15 min to label the nucleus. Finally, cells were washed with PBS for three times and observed by a confocal microscope.^4-5^

**Cytotoxicity assay**

4T1 cells and GES-1 cells were respectively seeded into 96-well plates with the density of 1 × 10^4^ cells for each well and incubated for 12h, then treated with different concentrations (0, 50, 100, 150 and 200 μg/mL, as an equivalent dosage of ICG) of different kinds of nano-platform (ZIF-8/ICG, MnO_2_/ZIF-8/ICG, and FA-EM@MnO_2_/ZIF-8/ICG) with NIR irradiation, or with PBS and PBS + NIR as control. After 48 h incubation, cell counting kit-8 (CCK-8) were utilized to analysis the cells viability.^6-7^

**Histological analysis**

To explore the therapeutic effects and the biocompatibility of FA-EM@MnO_2_/ZIF-8/ICG, the tumors and major organs (heart, liver, spleen, lung and kidney) of the mice were dissected and sectioned for transferase-mediated deoxyuridine triphosphate nick end labeling (TUNEL) staining and H&E staining.^8^ All the histological was carried out by Servicebio (Wuhan, China).

**Results**


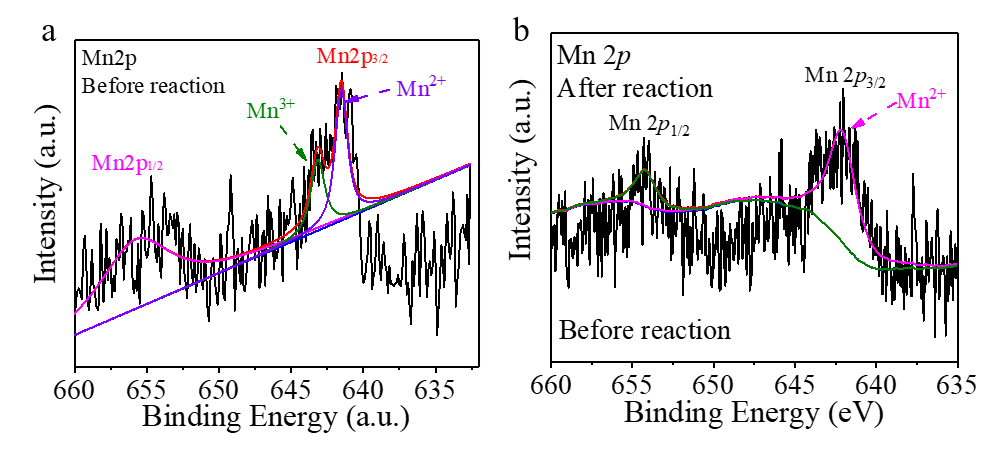


**Fig S1.** Narrow XPS scan spectra of Mn2p in FA-EM@MnO_2_/ZIF-8/ICG (a) and FA-EM@MnO_2_/ZIF-8/ICG in TME-simulating solution (b).


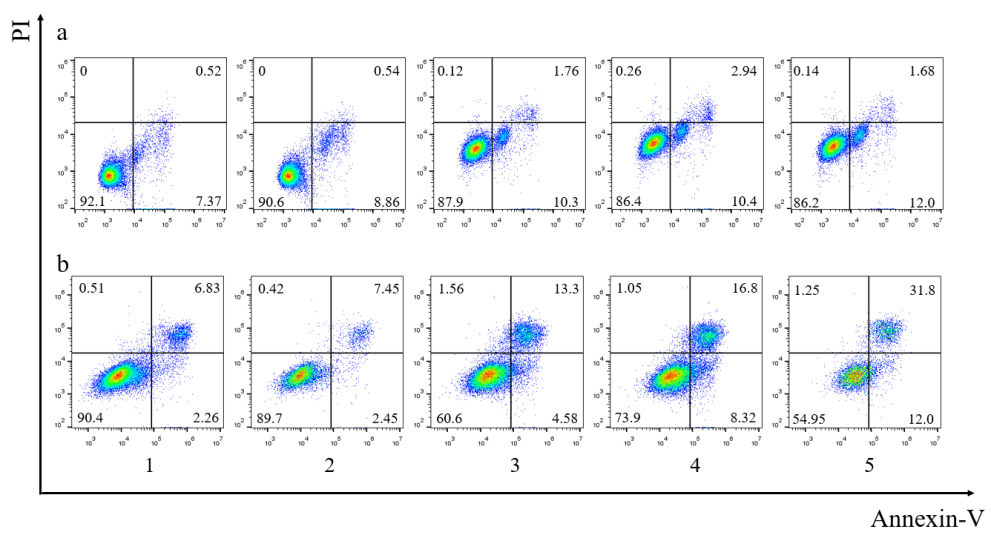


**Fig S2.** Viability of GES-1 cells (a) and 4T1cells (b) upon treated with PBS (1), PBS + NIR (2), ZIF-8/ICG + NIR (3), MnO_2_/ZIF-8/ICG + NIR (4), and FA-EM@MnO_2_/ZIF-8/ICG + NIR (5) by flow-cytometry.


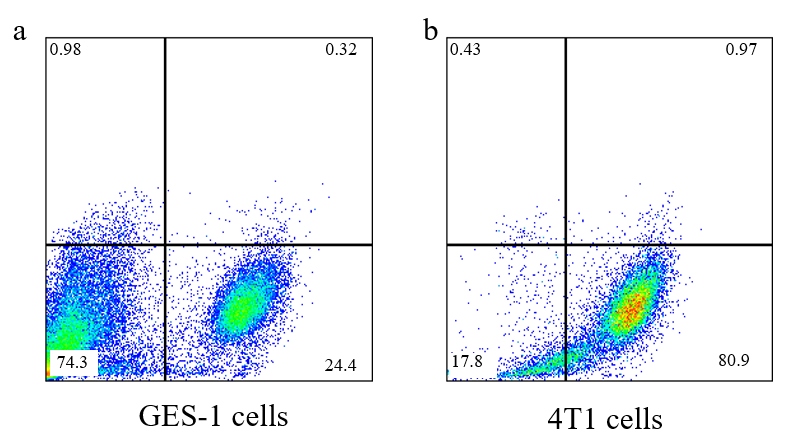


**Fig S3.** Flow-cytometry analyzed 4T1 and GES-1 cells uptake behavior for FA-EM@MnO_2_/ZIF-8/ICG nano-platform.


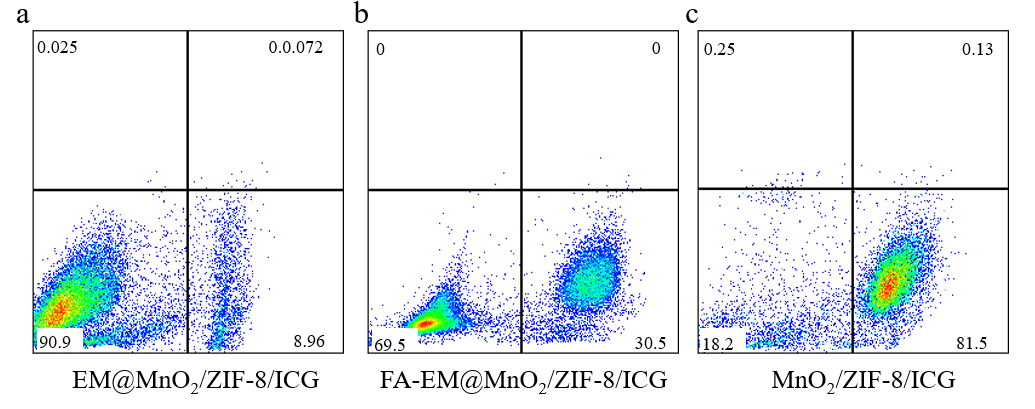


**Fig S4.** Flow-cytometry analyzed macrophage cells phagocytosis behavior with EM@MnO_2_/ZIF-8/ICG (a), FA-EM@MnO_2_/ZIF-8/ICG (b), and MnO_2_/ZIF-8/ICG (c) nano-platform.


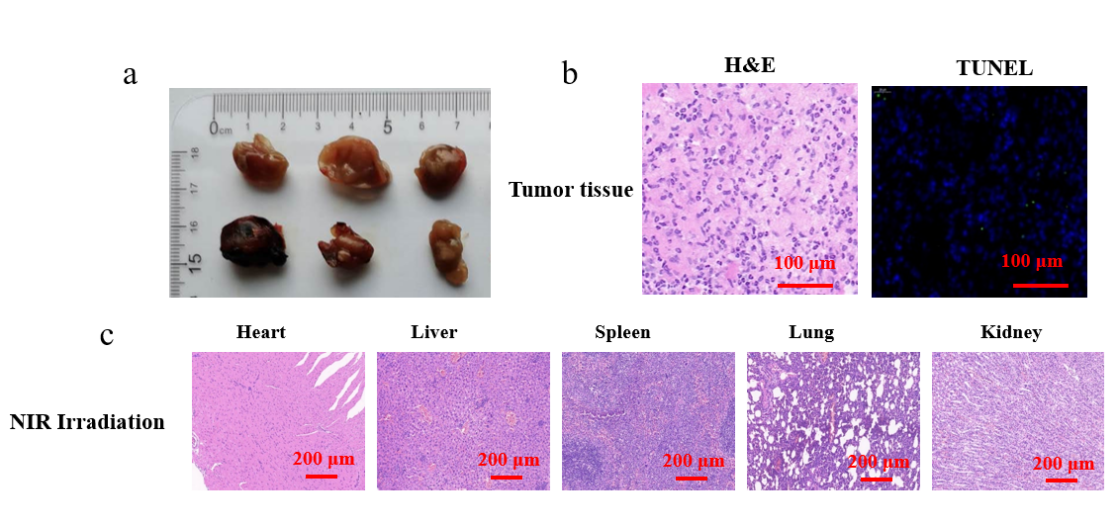


**Fig S5.** (a) Photographs of the tumors on day 15 post-injection after the last treatment of pure NIR irradiation. (b) H&E (left) and TUNEL staining (right) of tumor slices collected from 4T1 tumor-bearing mice after treatment of pure NIR irradiation. Scale bars: 100 μm. (c) H&E staining of various organs collected from 4T1 tumor-bearing mice after treatments of pure NIR irradiation. Scale bars: 200 μm.


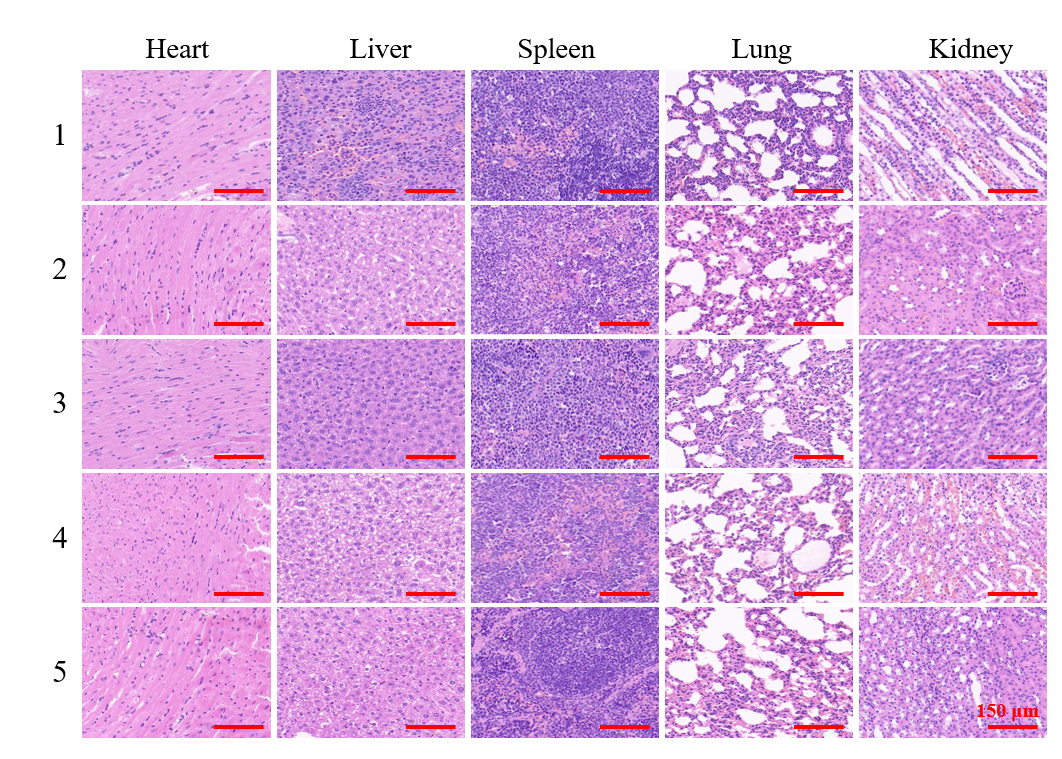


**Fig S6.** H&E staining of various organs collected from 4T1 tumor-bearing mice after different treatments of PBS (1), PBS + NIR (2), ZIF-8/ICG + NIR (3), MnO_2_/ZIF-8/ICG + NIR (4), FA-EM@MnO_2_/ZIF-8/ICG + NIR (5). Scale bars: 150 μm.

**References**

1. Sun, Q.; Bi, H.; Wang, Z.; Li, C.; Wang, C.; Xu, J.; Yang, D.; He, F.; Gai, S.; Yang, P., O_2_-Generating Metal-Organic Framework-Based Hydrophobic Photosensitizer Delivery System for Enhanced Photodynamic Therapy. *ACS Appl. Mater. Interfaces,* 2019. 11: 36347-36358.

2. Fang, Z.; Li, X.; Xu, Z.; Du, F.; Wang, W.; Shi, R.; Gao, D., Hyaluronic acid-modified mesoporous silica-coated superparamagnetic Fe_3_O_4_ nanoparticles for targeted drug delivery. *Int. J. Nanomedicine,* 2019. 14: 5785-5797.

3. Min, H.; Wang, J.; Qi, Y.; Zhang, Y.; Han, X.; Xu, Y.; Xu, J.; Li, Y.; Chen, L.; Cheng, K.; Liu, G.; Yang, N.; Li, Y.; Nie, G., Biomimetic Metal-Organic Framework Nanoparticles for Cooperative Combination of Antiangiogenesis and Photodynamic Therapy for Enhanced Efficacy. *Adv. Mater.,* 2019. 31: e1808200.

4. Zeng, F.; Qin, H.; Liu, L.; Chang, H.; Chen, Q.; Wu, L.; Zhang, L.; Wu, Z.; Xing, D., Photoacoustic-immune therapy with a multi-purpose black phosphorus-based nanoparticle. *Nano Res.,* 2020. 13: 3403-3415.

5. Zhang, Z.; Ni, D.; Wang, F.; Yin, X.; Goel, S.; German, L. N.; Wang, Y.; Li, J.; Cai, W.; Wang, X., In vitro study of enhanced photodynamic cancer cell killing effect by nanometer-thick gold nanosheets. *Nano Res.,* 2020. 13: 3217-3223.

6. Xiang, H.; Lin, H.; Yu, L.; Chen, Y., Hypoxia-Irrelevant Photonic Thermodynamic Cancer Nanomedicine. *ACS Nano,* 2019. 13: 2223-2235.

7. Chen, Y.; Xiang, H.; Zhuang, S.; Shen, Y.; Chen, Y.; Zhang, J., Oxygen-Independent Photocleavage of Radical Nanogenerator for Near-IR-Gated and H_2_O-Mediated Free-Radical Nanotherapy. *Adv. Mater.,* 2021. 33: e2100129.

8. Liu, B.; Hu, F.; Zhang, J.; Wang, C.; Li, L., A Biomimetic Coordination Nanoplatform for Controlled Encapsulation and Delivery of Drug-Gene Combinations. *Angew. Chem. Int. Ed.,* 2019. *58*: 8804-8808.
